# A Neurodisparity Index of Nationwide Access to Neurological Health Care in Northern Ireland

**DOI:** 10.3389/fneur.2021.608070

**Published:** 2021-02-12

**Authors:** Mark O. McCarron, Mike Clarke, Paul Burns, Michael McCormick, Peter McCarron, Raeburn B. Forbes, Luke V. McCarron, Fiona Mullan, Ferghal McVerry

**Affiliations:** ^1^Department of Neurology, Altnagelvin Hospital, Derry, Ireland; ^2^HSC Statistical and Methodological Support Service, Queen's University Belfast, Belfast, Ireland; ^3^Department of Neuroradiology, Royal Victoria Hospital, Belfast, Ireland; ^4^Acute Stroke Unit, Craigavon Hospital, Portadown, Ireland; ^5^The Drug Centre, Dublin, Ireland; ^6^Department of Neurology, Craigavon Hospital, Portadown, Ireland; ^7^University of Cambridge School of Clinical Medicine, Cambridge, United Kingdom

**Keywords:** stroke, multiple sclerosis, disparities, thrombolysis (tPA), mechanical thrombectomy, disease modifying drug

## Abstract

Nationwide disparities in managing neurological patients have rarely been reported. We compared neurological health care between the population who reside in a Health and Social Care Trust with a tertiary neuroscience center and those living in the four non-tertiary center Trusts in Northern Ireland. Using the tertiary center Trust population as reference, neurodisparity indices (NDIs) defined as the number of treated patients resident in each Trust per 100,000 residents compared to the same ratio in the tertiary center Trust for a fixed time period. NDIs were calculated for four neurological pathways—intravenous thrombolysis (iv-tPA) and mechanical thrombectomy (MT) for acute ischemic stroke (AIS), disease modifying treatment (DMT) in multiple sclerosis (MS) and admissions to a tertiary neurology ward. Neurological management was recorded in 3,026 patients. Patients resident in the tertiary center Trust were more likely to receive AIS treatments (iv-tPA and MT) and access to the neurology ward (*p* < 0.001) than patients residing in other Trusts. DMT use for patients with MS was higher in two non-tertiary center Trusts than in the tertiary center Trust. There was a geographical gradient for MT for AIS patients and ward admissions. Averaged NDIs for non-tertiary center Trusts were: 0.48 (95%CI 0.32–0.71) for patient admissions to the tertiary neurology ward, 0.50 (95%CI 0.38–0.66) for MT in AIS patients, 0.78 (95%CI 0.67–0.92) for iv-tPA in AIS patients, and 1.11 (95%CI 0.99–1.26) for DMT use in MS patients. There are important neurodisparities in Northern Ireland, particularly for MT and tertiary ward admissions. Neurologists and health service planners should be aware that geography and time-dependent management of neurological patients worsen neurodisparities.

## Introduction

Notable advances in the diagnosis and treatment of disabling neurological conditions have been achieved in recent years. For example, a range of disease modifying treatments (DMTs) have been approved for primary progressive and relapsing forms of multiple sclerosis (MS), while reperfusion therapies such as intravenous thrombolysis (iv-tPA) and mechanical thrombectomy (MT) for acute ischemic stroke (AIS) substantially reduce a patient's risk of death and dependency (1, 2).

However, despite such advances, barriers to diagnosis and treatment could lead to inequity in patient benefits. Factors that are related to potential inequity include sex, ethnicity and socioeconomic position. The term neurodisparity has been used to highlight this inequity, which has existed across a range of neurological conditions for decades (3). Ultimately to minimize neurodisparities, neurology services (in general and tertiary hospitals) need to be able to recognize and respond proportionately and almost synchronously to emerging neuro-epidemiological findings and service demands.

The UK Royal Colleges of Physicians and the Association of British Neurologists have provided joint guidance on the development of local neurology services including those in acute general hospitals (4). There are recommendations for patients with complex and urgent neurological problems to be directly managed by neurologists in neurology or neuroscience centers (5), but there are few robust population assessments of such developments.

Northern Ireland is uniquely placed to measure disparities in health care for a number of reasons. It has a homogenous population predominantly of a single race with little variation in prevalence of common disorders including stroke (6) and little variation in socioeconomic distribution among the populations of the five Health and Social Care (HSC) Trusts (7). Furthermore, similar prevalence data for multiple sclerosis (MS) has been recorded in the Northern and Western HSC Trust regions (8, 9).

Benchmarking management of specific acute and chronic neurological disorders in general and tertiary hospitals has been infrequently documented (10). To investigate whether neurodisparity existed we compared the population provision of neurology health care in a Trust with a tertiary center to neurology health care in Trusts with general hospitals but no tertiary center. We examined delivery of therapeutic interventions for two common neurological disorders—AIS and MS, and the real-time use of a single regional neurology inpatient ward using a population-based neurodisparity index (NDI). We chose AIS and MS not only because they are common neurological disorders, but also because new therapies for these diseases, have emerged in recent decades allowing an assessment of health care responsiveness to relatively recent therapeutic developments.

## Methods

### Geography, Population and Health Care Services

Northern Ireland, one of the four countries of the United Kingdom (UK) has an area of 5,460 square miles and a population of 1.885 million citizens. Health care services are organized under five HSC Trusts (Supplementary Figure 1) providing secondary care (acute general hospital) facilities for individuals residing in a particular Trust area. Tertiary services, including a single neuroscience ward for neurological patients, are based in the Belfast HSC Trust (BHSCT) with readily available regional neuroimaging and neuroradiologists, neurophysiology (EMG/NCS), neurosurgery, neuropathology and neuropsychology. Patients requiring intensive neurological assessment or monitoring (e.g., for Guillain Barré syndrome or bulbar myasthenic crisis), or investigations or treatment, or who cannot be managed in other Trusts due to limited neurological resources are expected to be transferred to the regional neurology ward in the neuroscience center in the BHSCT (5).

### Thrombolysis for AIS

Intravenous thrombolysis for AIS is provided at one or more centers in each Trust (Supplementary Figure 1). Using prospectively collected data on AIS patients treated with iv-tPA, we assessed the number of patients treated per year with iv-tPA over the 3 years 2013 to 2015 inclusive in the tertiary center Trust (BHSCT) and the four non-tertiary center Trusts. Data was retrieved from medical notes within each Trust and Trust residence was recorded. We compared population-based treatment rates for residents in each Trust, using patients living in the tertiary neuroscience Trust as the reference-treated population. Further details of the hospital-treated populations have been published elsewhere (11).

### Mechanical Thrombectomy for AIS

Northern Ireland has one MT service located in the tertiary neuroscience Trust (BHSCT). Most patients living outside that Trust who need MT are transferred from their acute hospital setting to the neuroscience center in the BHSCT for the procedure (a so-called “drip and ship model”). From this prospectively collected database, we retrieved MT and residence data for consecutive patients for the 2 years January 1, 2018 to December 31, 2019 inclusive and compared the number of MT-treated patients residing in the tertiary neuroscience Trust with the number of MT-treated patients residing in each of the other Trusts. Unique patient identification numbers were matched to postcodes to derive each patient's Trust of residence. Evidence for clinical benefit from MT emerged in 2015. Stroke neurologists and neuroradiologists in Northern Ireland had participated in one of the pivotal trials demonstrating the safety and efficacy of MT (12).

### Disease-Modifying Therapy in Multiple Sclerosis

A prospectively updated database of patients on DMTs for MS has been developed centrally in Northern Ireland to oversee the prescribing and dispensing of these drugs. The central register is Trust-specific, with the addresses of MS patients being used to determine their Trust residency. Consultant neurologists provide clinics for patients with neurological disorders including MS, in 11 of 12 hospitals in Northern Ireland. There are at least two hospitals in each HSCT with neurology clinics. There is also a tertiary Trust multidisciplinary MS clinic to advise and assess patients for DMT. We performed a service evaluation to determine the number of residents with MS who were receiving a DMT in 2016 in each Trust.

### Inpatients in Regional Neurology Ward

There is one 17-bed neurology ward in the regional neuroscience center Trust for the population of 1.885 million people of Northern Ireland. The staff on the neurology ward includes trainee neurologists, dedicated neurology nurses and allied health professionals. Criteria for admission include conditions for which neurology care is known to improve outcome (e.g., Guillain Barré syndrome and encephalitis) (5). Neurosurgery, neurophysiology, neuropathology and neuropsychology services are only available in the neuroscience hospital in this Trust. Within the tertiary Trust we retrieved daily inpatient records of patient occupancy of the tertiary neurology ward with address postcodes to determine the Trust residence of inpatients. With BHSCT audit approval, a proforma was competed to include age, sex and duration of stay over a 3 month period November 2018 to January 2019 inclusive.

### Statistical Analyses

We derived the number of residents in each Trust from Northern Ireland Statistics and Research Agency annual data (13). We used these data and the data described above for the management of neurological conditions to determine neurodisparity using a novel neurodisparity index (NDI) defined as the ratio of the number of patients treated in a non-tertiary center Trust/100,000 population/time unit compared to the number of patients treated in the tertiary center Trust/100,000 population/same time unit.

The closer the NDI is to one, the less inequity in the provision of that specific type of health care. NDIs were calculated for the following:

- MS patients on any DMT
- patients receiving reperfusion therapy for AIS (iv-tPA and MT)
- access and length of stay to the dedicated regional inpatient neurology ward.

Where data acquisition spanned more than 1 year, the population for the midpoint in the time period was used to calculate the NDI. We reported comparisons of treatments and inpatient stays between the tertiary neuroscience Trust (BHSCT) and the four other Trusts, both individually and combined, per 100,000 population for each Trust area, and calculated 95% confidence intervals (95%CI) for a proportion using Wilson's method. The 95% CI for the NDI were generated from two rates. Chi square statistics for proportions were calculated from categorical data. Statistical analyses were performed with Open Source Epidemiologic Statistics for Public Health (http://www.openepi.com/Menu/OE_Menu.htm).

## Results

In the study, a total of 3,026 patient events were recorded. These included 846 (28%) AIS patients treated with iv-tPA, 228 (7%) AIS patients treated with MT, 1,840 (61%) patients with MS on a DMT and 112 (4%) patients admitted to the tertiary neurology ward for further management. The different hospital centers for each of these four interventions are shown on the map of Northern Ireland (Supplementary Figure 1).

### AIS—Intravenous Thrombolysis Treatment

**Figure 1:**
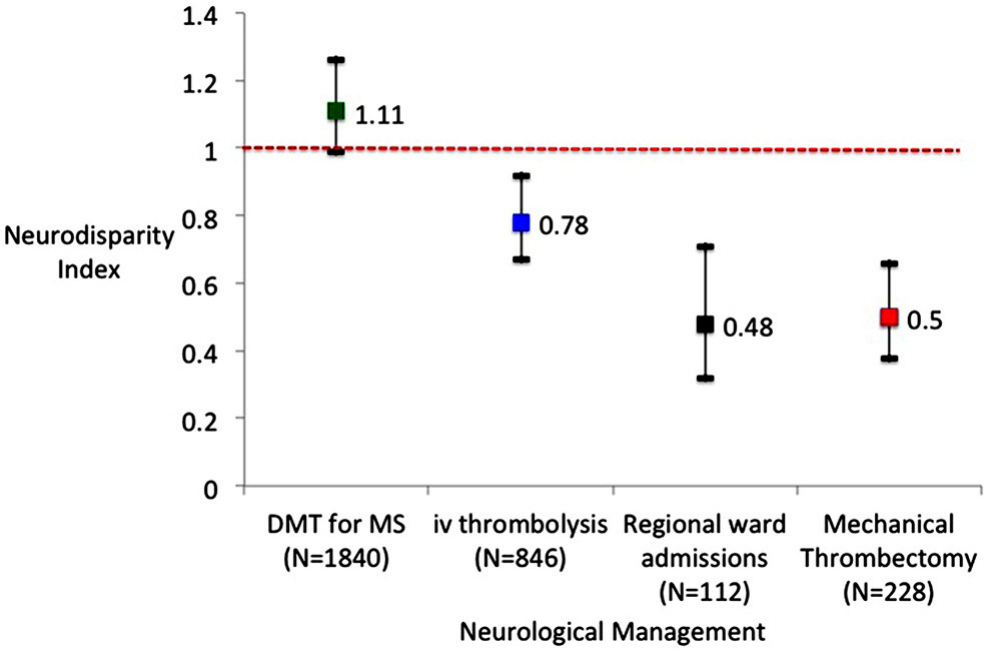
Neurodisparitiy indices for four Health and Social Care Trusts compared to the tertiary Trust for disease modifying therapy (DMT) for multiple sclerosis (MS), intravenous thrombolysis for acute ischemic stroke, admissions to the regional neurology ward and mechanical thrombectomy for acute ischemic stroke.

Over the 3 years 2013 to 2015 inclusive, 846 patients were treated with iv-tPA across Northern Ireland. Population-based treatment with iv-tPA for AIS was highest in the Trust with the tertiary neuroscience center (18.5 patients treated/100,000/year, 95%CI 16.8–20.2). Treatment rates in the four other Trusts were lower, ranging from 14.4 to 14.7 patients treated/100,000/year (Table 1). On average the non-tertiary Trust patients were significantly less likely to be treated with iv-tPA (14.5/100,000/year (95%CI 13.4–15.7) than those in the BHSCT. The summary NDI for iv-tPA for all non-tertiary Trust residents relative to the neuroscience center Trust was 0.78 (95%CI 0.67–0.92, Figure 1).

**Table 1:** Health and Social Trust neurodisparity indices for thrombolytic therapy and mechanical thrombectomy in acute ischemic stroke in Northern Ireland.

**Trust**	**Number of patients treated**	**Number of patients treated per 100,000/year (95%CI)**	**Neurodisparity index (95%CI)**
**Intravenous thrombolytic therapy**			
BHSCT	196	18.5 (16.8–20.2)	Reference
NHSCT	204	14.5 (13.0–16.0)	0.79 (0.64–0.98)
SEHSCT	156	14.7 (13.0–16.5)	0.78 (0.64–0.97)
SHSCT	161	14.5 (12.9–16.2)	0.78 (0.62–0.97)
WHSCT	129	14.4 (12.6–16.3)	0.78 (0.67–0.92)
**Mechanical thrombectomy**			
BHSCT	73	10.2 (7.4–14.1)	Reference
NHSCT	45	4.9 (3.2–7.3)	0.46 (0.32–0.67)
SEHSCT	42	5.8 (3.8–8.9)	0.57 (0.39–0.83)
SHSCT	47	6.2 (4.1–9.2)	0.60 (0.41–0.86)
WHSCT	21	3.5 (1.9–6.3)	0.34 (0.21–0.55)

*BHSCT, Belfast Health and Social Care Trust; NHSCT, Northern Health and Social Care Trust; SEHSCT, Southeastern Health and Social Care Trust; SHSCT, Southern Health and Social Care Trust; WHSCT, Western Health and Social Care Trust*.

### AIS—Mechanical Thrombectomy

A total of 228 patients in Northern Ireland had MT over the 2 years 2018 to 2019. The WHSCT, which is the furthest away from BHSCT, had the lowest NDI for MT at 0.36 (95%CI 0.18–0.69). The Southern Health and Social Care Trust (SHSCT) had an NDI of 0.62, the highest of the non-tertiary Trusts and the 95%confidence intervals included 1.0. MT procedure rates were approximately two-fold higher for patients living in the tertiary Trust (BHSCT) than for patients living in any other Trust, with an NDI of 0.50 (95%CI 0.38–0.66) for the four non-tertiary Trusts (Figure 1).

### Disease Modifying Therapy for Multiple Sclerosis

In 2016, 1,840 patients were on a DMT for MS (Table 2). The treatment rate per population ranged from 86.9/100,000 population in the SHSCT to 114.4/100,000 population in the South Eastern Health and Social Care Trust (SEHSCT). The BHSCT had 90.5 MS patients on a DMT/100,000 population. The NDIs ranged from 0.96 to 1.26 across the Trusts, with two Trusts (NHSCT and SEHSCT) having higher NDIs for DMTs in MS than the tertiary Trust (BHSCT). The NDI comparing BHSCT with the other four Trusts for DMT use was 1.11 (95%CI 0.99–1.26, Figure 1).

**Table 2:** Health and Social Trust neurodisparity indices for disease modifying treatments for multiple sclerosis in Northern Ireland.

**Trust**	**Number of MS patients on DMT**	**Number treated/100,000 (95% CI)**	**Neurodisparity index (95%CI)**
BHSCT	321	90.5 (81.1–100.9)	Reference
NHSCT	502	106.1 (97.2–115.8)	1.17 (1.02–1.35)
SEHSCT	408	114.4 (103.8–126.0)	1.26 (1.09–1.46)
SHSCT	328	86.9 (78.0–96.9)	0.96 (0.82–1.12)
WHSCT	281	93.5 (83.2–105.1)	1.03 (0.88–1.21)

*DMT, disease modifying treatment; BHSCT, Belfast Health and Social Care Trust; NHSCT, Northern Health and Social Care Trust; SEHSCT, Southeastern Health and Social Care Trust; SHSCT, Southern Health and Social Care Trust; WHSCT, Western Health and Social Care Trust*.

### Neurology Ward Occupancy in Tertiary Center

Table 3 shows that inpatients in the neurology ward in the tertiary neuroscience center were more likely to have their primary residence in the tertiary center Trust (BHSCT) or the adjacent Trust (SEHSCT). The Trusts not bordering the BHSCT (WHSCT and SHSCT Supplementary Figure 1) had the lowest NDIs for patient admissions to the tertiary neurology ward. The NDI for regional ward admissions among the non-tertiary center Trusts was 0.48 (95%CI 0.32–0.71, Figure 1). The number of occupied bed-days per Trust resident yielded low NDIs for Trusts geographically located further away from the tertiary center Trust (Supplementary Table 1).

**Table 3:** Neurodisparity indices for the number of patients in the regional neurology ward per Health and Social Care Trust.

**Trust**	**Number**	*P*-value^*^	**Number per 100,000 (95% CI)**	**Neurodisparity index (95% CI)**
BHSCT	37	Reference	10.3 (7.5–14.3)	Reference
NHSCT	26	0.016	5.5 (3.7–8.1)	0.53 (0.32–0.87)
SEHSCT	30	0.44	8.3 (5.8–11.8)	0.80 (0.50–1.30)
SHSCT	7	<0.001	1.8 (0.9–3.8)	0.18 (0.07–0.38)
WHSCT	12	0.004	4.0 (2.3–6.9)	0.38 (0.20–0.74)

* *Chi square test. BHSCT, Belfast Health and Social Care Trust; NHSCT, Northern Health and Social Care Trust; SEHSCT, Southeastern Health and Social Care Trust; SHSCT, Southern Health and Social Care Trust; WHSCT, Western Health and Social Care Trust*.

## Discussion

This study has highlighted both disparities and equity in neurological hospital services in Northern Ireland, a country in the UK with a very homogenous population. Time-dependent management and geographical factors were particularly relevant for regional neurology ward admissions and the use of MT for AIS patients with large vessel occlusions. These factors influenced access to specialized management of specific neurological disorders. Better equity of time-dependent treatment existed for iv-tPA for AIS among eight centers geographically spread across Northern Ireland. In addition, elective, non-acute management of MS (i.e., use of DMT) demonstrated the best overall equity of access to neurological health care. This may be because patients with MS who did not require urgent treatment were able to access DMTs from many neurology clinics geographically spread across Northern Ireland. Two Trusts even had a higher rate of DMT use than the tertiary reference tertiary center Trust.

Neurology health care within the National Health Service in the UK comprises heterogeneous services working closely with neuroscience centers for inpatient services, allied with investigations and supporting neuroscience staff (5). In Northern Ireland, a single neuroscience center has been established to support acute general hospitals, which have variable local neurology services. There is no dedicated neurology center or ward other than in the neuroscience center in the BHSCT. As in the UK in general, there has been, until recently, little scrutiny or real-time monitoring of the impact and equity of these heterogenous regional and national hospital-based neurology services (5). It is estimated that even in the better resourced areas of the UK, less than half of all patients for whom evidence suggests better outcomes from management by a neurologist (encephalitis, epilepsy, MS, Guillain Barré syndrome, myasthenia gravis), are admitted to hospital under neurology (5). In England, there is a ten-fold variation in this parameter and a longer length of stay in hospital among patients not under the direct care of neurologists, although this variation was not related to the number of neurologists in a region (5). In the UK as a whole, there is, on average, only one neurologist per 79,000 people, and even less coverage in Northern Ireland (21.5 whole time equivalent neurologists for a population of 1.885 million or 1 per 88,000). These data and the findings of our study emphasize the potential for improvement in the organization of neurology care in Northern Ireland.

It has been acknowledged that the current UK variation in neurology health care results in “an inequitable patchwork of provision,”(14) exacerbated by a lack of guidance on how neuroscience centers should deliver neurology health care (5). One model of a neurology network has emerged in Liverpool (Walton Center Foundation Trust), which has provided some evidence of an equitable, sustainable and deliverable model of a neurology service for a population of 2.7 million people (14). The Liverpool model has successfully overcome the inverse care law (15) for emergency admissions to their neuroscience center, demonstrating on a population basis, that neurological emergencies were just as likely to be admitted to a ward in the neuroscience center whether they came from remote or local hospitals (14). This system (a stand-alone neuroscience center in a separate hospital with regional remit) avoids the conflict between regional specialized care and local care for all neurological patients in the one hospital.

One potential limitation of our study is the use of a novel NDI, which assumes similar epidemiology for acute neurological disorders (such as AIS) and MS in each of the five Trusts in Northern Ireland. Each Trust has 300,000 to 500,000 residents and one of the strengths of basing this study in Northern Ireland is the homogenous and stable population of this country with consistent disease epidemiology (6, 8, 9). Therefore, the crude (unadjusted) NDI calculations in the current study may be justified by the small size of the Northern Ireland population (1.885 million). Although some determinants of health (obesity and cigarette smoking) are equally distributed among the Trusts, an important social determinant of health, absolute poverty was present in 27% of WHSCT residents compared to a Northern Ireland average of 16% (Supplementary Table 2), suggesting that geography and poverty may influence access to time-critical neurological management. However, when seeking to generalize the application of NDIs, correction for factors such as disease incidence, age, sex and socioeconomic influences using individual level data (16) may need to be incorporated in order to calculate standardized NDIs.

In contrast to previous studies of stroke services in Northern Ireland, our study is population-, rather than hospital-based (11, 17, 18). And, although we have used data for slightly different time periods (due to a lack of contemporaneous data collection periods), there has been little evidence of temporal variation, reflecting the stability of both the Northern Ireland population and its health services. The MT rate in Northern Ireland compares well with that in many other European countries at approximately 60/million population/year. Northern Ireland would be ranked 15 out of 44 countries using 2016/2017 data (19). While much variation exists in stroke services between European countries, there is an expectation of equal access to all medical services within a country. MT rates in Northern Ireland are more than eight times above that of the UK for a similar time period, reflecting the current lack of a comprehensive nationwide thrombectomy service throughout Great Britain (19). It will be important to measure temporal trends for MT not only within the Northern Ireland population but also to maintain comparisons with other European countries. The Northern Ireland intravenous thrombolysis rate of 150/million population/year is similar to the rest of the UK, ranking 17 among 44 European countries (19).

Stroke neurologists know that there is a treatment gap for patients living in rural and remote areas (20–22). Some of the stroke neurodisparity may be reduced with technical solutions such as helicopter transfer to a MT center (23) or a mobile stroke unit with telemedicine connection (24). The current organization of stroke services in Northern Ireland, where dedicated acute stroke units are provided in each Trust, has yielded better (but still not perfect) equity for iv-tPA treatment for AIS than for the single center MT, as shown by the geographical inequity gradient for MT. Looking to the future, stroke research in Northern Ireland and elsewhere is actively pursuing ways to streamline time-dependent treatments to minimize delays and neurodisparities for geographically-remote patients (25, 26).

Structural disparities in health care in other countries, such as the USA, have been shown to influence outcomes. Racial and geographic disparities in breast cancer mortality (27) and pregnancy-related deaths (28) exist. Furthermore, recognition of racial, ethnic and geographic issues in the delivery of neurological health care has prompted the setting up of an American Academy of Neurology Taskforce to tackle these disparities (29). While neurodisparity is often recognized as an issue related to economics or ethnicity, vigilance and critical monitoring of service provision can prompt improvements in access to treatment of neurological patients in geographically remote areas (30).

The observation of neurodisparities in our study should alert health service commissioners, epidemiologists, clinicians and the public to the need to monitor hospital health care for the presence of inequity. We recommend a continuous population-based data monitoring process. Neurodisparities might then be minimized with appropriately evidence-based interventions. Research into the evaluation of interventions should highlight efficiency targets (31), so that improvements in NDIs correspond to more equitable enhanced local and national neurology services. This vigilance, it may be argued, should be an ethical obligation for patient safety (32).

## Data Availability Statement

The raw data supporting the conclusions of this article will be made available by the authors, without undue reservation.

## Ethics Statement

Ethical review and approval was not required for the study on human participants in accordance with the local legislation and institutional requirements. Written informed consent for participation was not required for this study in accordance with the national legislation and the institutional requirements.

All data from Trusts were anonymized for final analyses comparing Trust performance. The quality improvement departments of all five Trusts provided approval for the service evaluation for AIS patients. The quality improvement department of the WHSCT provided approval for the service evaluation for patients with MS (all data anonymized at source). The BHSCT audit department provided approval for the evaluation of the BHSCT inpatient neurology ward service.

## Author Contributions

MOM and FMc conceived the study, wrote the first draft, and approved the paper for submission. PB, MM, and FMu collected the data and revised drafts. MC, RF, PM, and LM contributed to the methodology and revised drafts of the paper. All authors contributed to the article and approved the final version for submission.

## Conflict of Interest

The authors declare that the research was conducted in the absence of any commercial or financial relationships that could be construed as a potential conflict of interest.

## Supplementary Material

Supplementary Figure 1 Map of the five Health and Social Care Trusts in Northern Ireland with neurological health care facilities.

Supplementary Table 1 Neurodisparity indices for the number of inpatient bed-days in the regional neurology ward per Health and Social Care Trust.

Supplementary Table 2 Distribution of some determinants of health among Health and Social Care Trust residents (adapted from www.nisra.gov.uk).
