# Supplementary figures and images for: A Neurodisparity Index of Nationwide Access to Neurological Health Care in Northern Ireland
Source: Front Neurol. 2021 Feb 12;12:608070. doi: 10.3389/fneur.2021.608070 (PMC7907594; doi:10.3389/fneur.2021.608070)

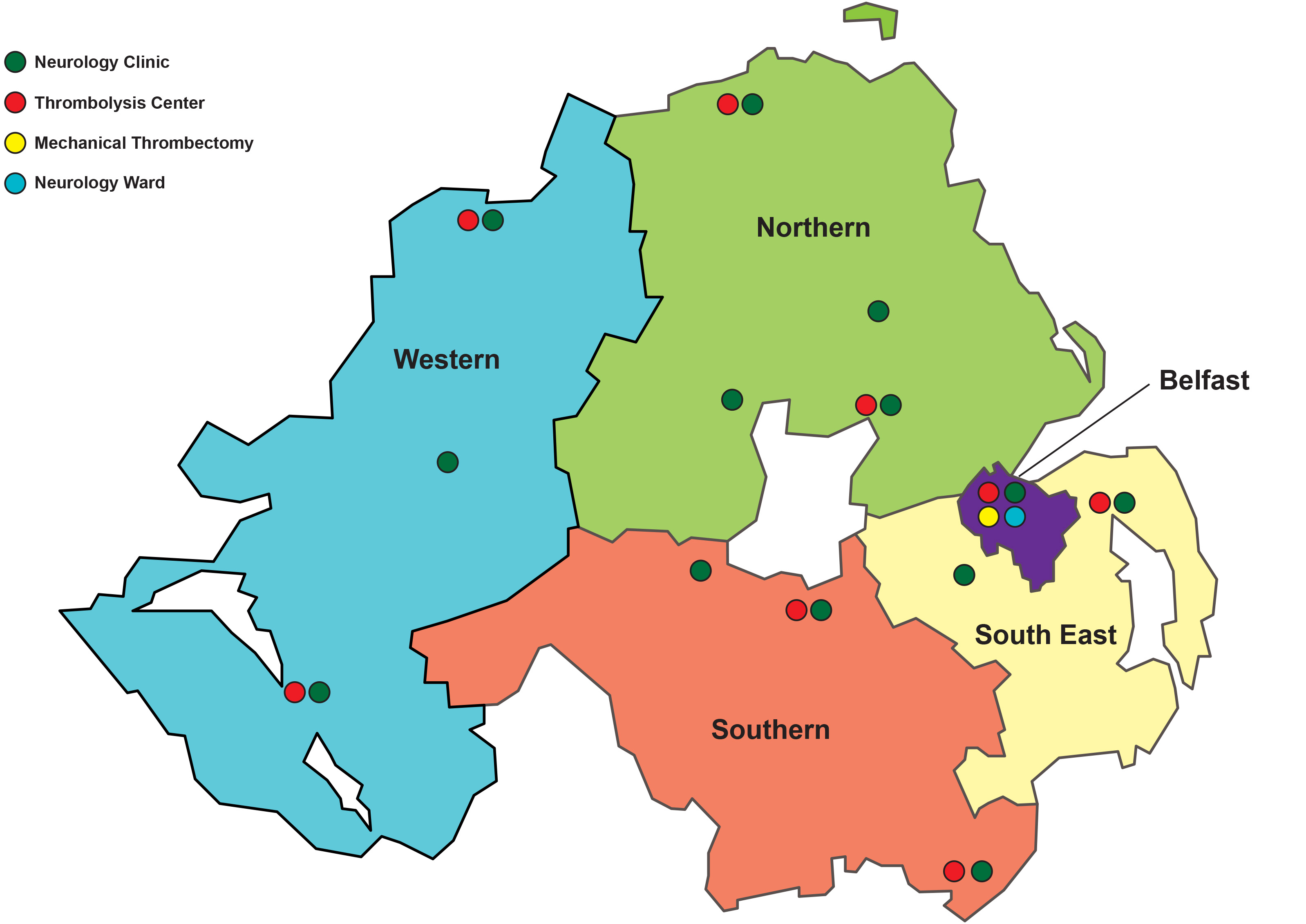

Supplement: Supplementary Figure 1 — Map of the five Health and Social Care Trusts in Northern Ireland with neurological health care facilities. [file Image_1.JPEG]
