# Supplementary material for: A Neurodisparity Index of Nationwide Access to Neurological Health Care in Northern Ireland
Source: Front Neurol. 2021 Feb 12;12:608070. doi: 10.3389/fneur.2021.608070 (PMC7907594; doi:10.3389/fneur.2021.608070)
Supplement: Supplementary Table 1 — Neurodisparity indices for the number of inpatient bed-days in the regional neurology ward per Health and Social Care Trust. [file Table_1.docx]

**Supplementary Table 1. Neurodisparity indices for the number of inpatient bed-days in the regional neurology ward per Health and Social Care Trust**

| **Trust** | **Inpatient days/3 months** | **Inpatient days/100,000 (95%CI)** | **Neurodisparity Index (95%CI)** |
| --- | --- | --- | --- |
| BHSCT | 525 | 146.8 (134.8-159.9) | Reference |
| NHSCT | 606 | 127.1 (117.3-137.6) | 0.87 (0.77-0.97) |
| SEHSCT | 434 | 120.1 (109.3-131.9) | 0.81 (0.72-0.93) |
| SHSCT | 141 | 36.8 (31.2-43.3) | 0.25 (0.21-0.30) |
| WHSCT | 108 | 35.7 (29.6-43.1) | 0.24 (0.20-0.30) |
| Non-BHSCT | 1289 | 84.6 (80.1-89.3) | 0.58 (0.52-0.64) |

BHSCT-Belfast Health and Social Care Trust, NHSCT-Northern Health and Social Care Trust, SEHSCT-Southeastern Health and Social Care Trust, SHSCT-Southern Health and Social Care Trust, WHSCT-Western Health and Social Care Trust.

**Supplementary Table 2. Distribution of some determinants of health among Health and Social Care Trust residents (adapted from www.nisra.gov.uk)**

| **Trust** | **Obesity (%)** | **Cigarette smoking (%)** | **Absolute poverty (%)** | **No qualifications* (%)** |
| --- | --- | --- | --- | --- |
| NI | 25 | 18 | 16 | 29 |
| BHSCT | 23.5 | 20.5 | 17 | 29.1 |
| NHSCT | 27.6 | 17.6 | 18 | 29 |
| SEHSCT | 26.1 | 15.2 | 11 | 24.5 |
| SHSCT | 23 | 20.4 | 12 | 30 |
| WHSCT | 23 | 19.7 | 27 | 33.7 |

2018 data except *data from 2011 census. NI-Northern Ireland, BHSCT-Belfast Health and Social Care Trust, NHSCT-Northern Health and Social Care Trust, SEHSCT-Southeastern Health and Social Care Trust, SHSCT-Southern Health and Social Care Trust, WHSCT-Western Health and Social Care Trust.
